# Low Infection Rate After Transrectal Implantation of Gold Anchor ™ Fiducial Markers in Prostate Cancer Patients After Non-broad-spectrum Antibiotic Prophylaxis

**DOI:** 10.7759/cureus.3526

**Published:** 2018-10-31

**Authors:** Enrique Castellanos, Peter Wersäll, Aris Tilikidis, Arja H Andersson

**Affiliations:** 1 Radiation Oncology, Karolinska University Hospital and Karolinska Institute, Stockholm, SWE; 2 Medical Physics, Karolinska University Hospital and Karolinska Institute, Stockholm, SWE

**Keywords:** prostate cancer, radiation therapy, infection, urinary tract infections (uti), complications, fiducial marker, radiation treatment, antibiotic prophylaxis, prostate sbrt, migration

## Abstract

Background

In 621 consecutive prostate cancer patients, the frequency of urinary tract infections (UTI) and marker loss was evaluated. They prophylactically received a single dose of non-broad-spectrum antibiotics and transrectal implantation of three thin needle fiducial markers, Gold Anchor ™ (GA).

Methods

The occurrence of UTIs, sepsis, hospitalization due to infection, and marker loss after implantation was assessed from the medical records containing notes from physicians and nurses from the day of implantation to the end of 29 fractions.

Results

UTIs occurred in two (0.3%) of the 621 patients. Neither sepsis nor hospitalization was noted. Loss/drop-out of three markers was noted among 1,863 markers implanted.

Conclusion

The use of thin needles for the implantation of fiducials appears to reduce the rate of infection despite the use of a single dose of non-broad-spectrum antibiotics as prophylaxis. The marker construct appears to provide stability in the tissues.

## Introduction

To minimize the complications associated with the implantation of fiducial markers for image-guided radiotherapy, a fine needle marker called Gold Anchor ™ (GA) for kilovoltage (kV) imaging was developed by Dr. I. Naslund at Radiumhemmet, Department of Oncology, Karolinska University Hospital, Stockholm. This idea came from decades of good experience of fine needle aspiration cytology with 25-gauge (G) needles, developed inhouse by Dr. Sixten Franzén in the 1950s [1]. This study evaluates the number of infections after the implantation of the GA markers in 621 prostate cancer patients receiving radiation therapy.

## Materials and methods

We retrospectively studied the records of 621 consecutive prostate cancer patients who got GA implanted per rectum solely by one of the uro-oncologists (E. Castellanos) from January 2007 to January 2016 at our institution. Urinary tract infections (UTI), sepsis, and hospitalization, as well as notations of marker migration or loss, were analyzed.

Patients received neoadjuvant hormonal treatment for three months before external curative radiation treatment with 72.5 Gy in 29 fractions. The mean age of the patients was 72 years (range: 46-85 years).

Patients were told to use a Microlax® enema in the morning of the day of the procedure. A peroral single dose of Bactrim® Forte (sulfamethoxazole 1,600 mg and trimethoprim 320 mg) was given one hour before implantation to all patients. No pre-procedural rectal swabs, stool cultures, or preop urinary culture were obtained to guide the selection of antibiotics.

The GA markers implanted have a diameter of 0.28 or 0.40 mm, both available in 10- or 20-mm length. The markers are of pure gold with 0.5% pure iron in an alloy to enhance magnetic resonance imaging (MRI) visibility. They are preloaded in 25G (0.50 mm/0.020” outer diameter) or 22G (0.70 mm/0.028”) fine needles. This study employed 200-mm-long 22G needles, loaded with a 0.28 × 20-mm marker delivered in a single blister pack with the product code GA200-20. When the marker is pushed out of the needle with the stylet, it folds itself in the tissue into a ball shape. The diameter of the ball is greater than the diameter of the needle so that the expanded marker cannot move back into the needle tract when the needle is retracted.

In a lithotomy position, three GA markers per patient were implanted transrectally in prostate, placed in the right lobe in the base and apex and in the other lobe in the midgland to avoid marker overlapping during imaging for patient setup. The 22G marker needle has a diameter similar to that of a local anesthesia needle. Injection of the anesthetic would be more painful than implantation of the fiducials itself; therefore, no anesthesia was used. Consequently, only three thin punctures through the rectal wall were needed per patient. This procedure typically takes only a few minutes. Computed tomography (CT) and MRI scans for the treatment planning were typically obtained the same day or within two days after implantation. Patients were informed to report fever or any local side effects after implantation.

Orthogonal kV images were acquired at each fraction for positioning, and the patients received a daily follow-up of side-effects by the nurses and/or the uro-oncologists of the RT section. Data were stored in the Information Networks for Cancer (INCA) register, a Swedish IT platform to facilitate the follow-up and evaluation of the health outcomes and quality. Facts about UTI, sepsis, and hospitalization after implantation of GA have been derived from our Clinical Electronic Medical Records and the INCA register.

The criteria for UTI were an oral temperature above 38.0 °C and a positive urine culture. The criteria for sepsis were defined as a life-threatening organ dysfunction caused by a dysregulated host response to infection according to the Sepsis-3 definitions [2].

## Results

All the 621 patients were followed up for side effects from the day of implantation to the end of 29 fractions. Two of them, 0.3%, had UTI.

In 2012, a 67-year-old man had UTI symptoms on the third day after implantation and was treated with ciprofloxacin 500 mg × 2 for one week without improvement. The urine culture showed resistant *E. coli* to ciprofloxacin, and he was given Bactrim® Forte 1 × 2 for seven days. This patient had a prostate-specific antigen (PSA) level of 6.2, stadium T1c, Gleason score 3+3, and a prostate volume of 45 cm^3^.

In 2014, a 71-year-old man had UTI. He was successfully treated with ciprofloxacin 500 mg × 2 for one week. This patient had a PSA level of 4.5, stadium T1c, Gleason score 3+4, and a prostate volume of 24 cm^3^.

There was no sepsis and no hospitalization due to implantation. No loss of the 1,863 GA markers had been noted during the daily setup, except for three patients (0.16%) in which one of the three markers in each patient was implanted very close to a transurethral resection of the prostate (TURP) cavity.

## Discussion

**Figure 1:**
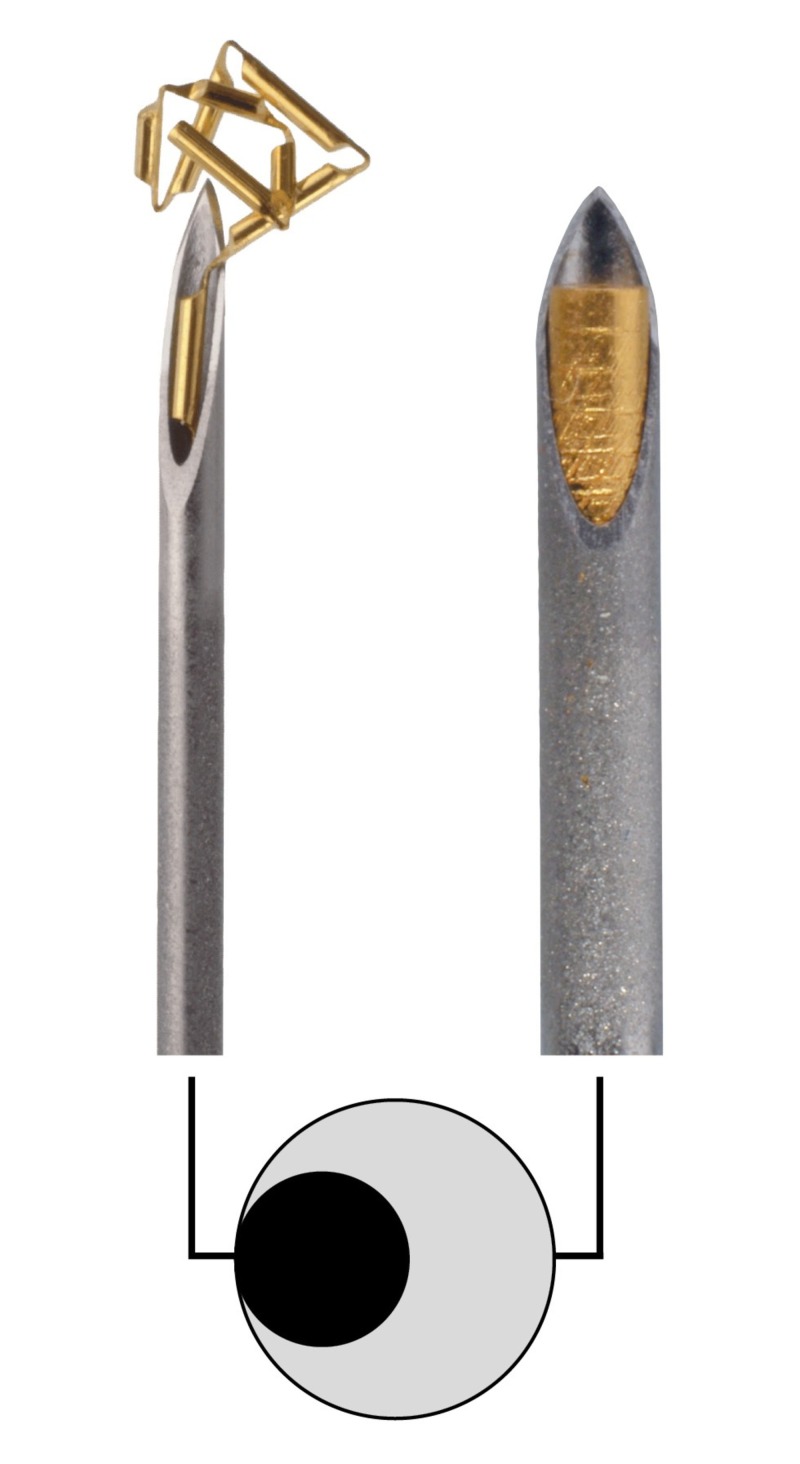
Comparison in diameter between Gold Anchor™ needle to the left and an 18G traditional needle for fiducial markers. The cross-sectional area is three to four times larger for 18G and 17G needles compared to the 22G thin needle of 0.7 mm for Gold Anchor

Traditionally, the fiducial markers had to be large enough to be visible on a megavoltage (MV) radiograph film or MV portal imaging devices. Therefore, it was a need to use 17-18G needles (1.47-1.25 mm/0.058-0.050” outer diameter) for the placement of fiducial markers. Despite improvements in the imaging technique, thick needles are still used for the implantation of large fiducial markers. The same type of thick needles, 17-18 G, are also used for prostate biopsies. Those needles have a three to four times larger cross-sectional area than the thin 22G needles used for GA in prostate (Figure 1).

**Figure 2:**
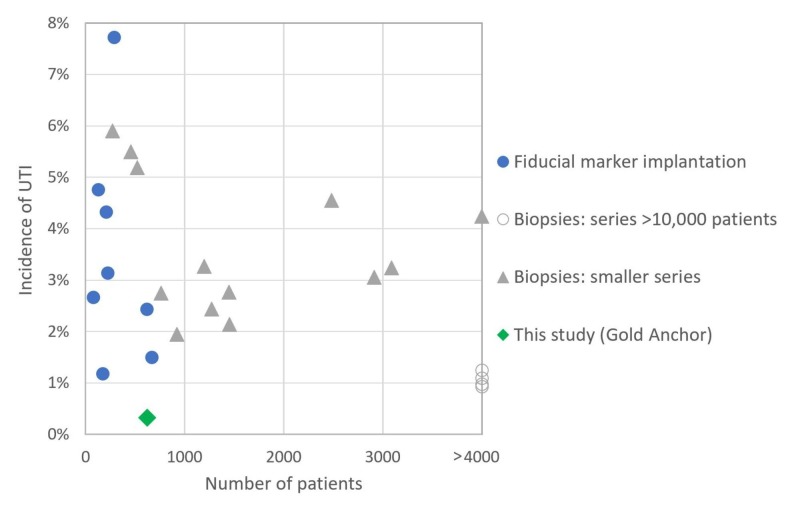
Incidence of UTI after transrectal biopsy or fiducial marker implantation

Figure 2 shows significant heterogeneity of the infection rates reported after transrectal biopsies or fiducial marker implantations [3-26].

Table 1 shows the infection rates reported among patients after transrectal fiducial marker implantation [3-10]. Table 2 shows the infection rates after transrectal prostate biopsies in a large series [10-13], and Table 3 shows the same in a small series [14-26]. The studies were conducted in various ways, in different time periods and with different antibiotic prophylaxis, without targeted antibiotics guided by rectal swab. This prevents a direct comparison of the results but gives an estimate of the infection rates.

**Table 1:** Incidence of UTIs after transrectal fiducial marker implantation UTI: urinary tract infection

Reference	Patients	UTI	UTI %
3	126	6	4.8%
4	223	7	3.1%
5	615	15	2.4%
6	75	2	2.7%
7	169	2	1.2%
8	285	22	7.7%
9	208	9	4.3%
10	666	10	1.5%
Sum	2,367	73	3.1%

**Table 2:** Incidence of UTIs after transrectal prostate biopsies in a large series UTI: urinary tract infection

Reference	Patients	UTI	UTI %
10	10,938	137	1.3%
11	212,065	2,333	1.1%
12	25,355	249	1.0%
13	59,469	553	0.9%
Sum	307,827	3,272	1.1%

**Table 3:** Incidence of UTIs after transrectal biopsies in a smaller series UTI: urinary tract infection

Reference	Patients	UTI	UTI %
14	1,273	31	2.4%
15	923	18	2.0%
16	1,446	40	2.8%
17	1,450	31	2.1%
18	521	27	5.2%
19	1.195	39	3.3%
20	764	21	2.7%
21	455	25	5.5%
22	3,087	100	3.2%
23	2,913	89	3.1%
24	2,484	113	4.5%
25	271	16	5.9%
26	9,241	392	4.2%
Sum	26,023	942	3.6%

A reasonable assessment of these studies is that more than 2% of the patients developed UTI after 17-18G needle procedures. Although there are fewer rectal wall penetrations during marker implantation than during biopsy, the rates of UTIs reported seem to be similar to the rates after biopsies. One possible explanation is the development of bacterial resistance related to the use of the same antibiotics as for the prostate biopsy.

*E. coli* infection after transrectal biopsy of the prostate has become significantly more common since 2000 due to a dramatic increase in fluoroquinolone-resistant *E. coli*. [13]. The World Health Organization (WHO) recommends that new antibiotics are reserved for serious infections and not used for prophylaxis. The use of ciprofloxacin, belonging to the WATCH group, used to treat cystitis (a type of UTI), should be dramatically reduced to avoid further development of resistance. Furthermore, the third group, RESERVE, includes antibiotics such as colistin and some cephalosporins that should be considered as last-resort options and used only in the most severe circumstances when all other alternatives have failed, such as for life-threatening infections due to multidrug-resistant bacteria [27]. The US Food and Drug Administration (FDA) recently advised that in the treatment of uncomplicated UTIs, fluoroquinolones should be reserved for those who lack other options due to other complications besides development of resistance [28].

## Conclusions

Infections after prostate biopsy and fiducial implantations with traditional 17G-18G needles are associated with the risk of UTI. In this study with 621 patients, we found a low UTI incidence after the transrectal implantation of Gold Anchor™ fine needle markers, despite the use of a non-broad-spectrum antibiotic prophylaxis. It is important to have a holistic view of all the inconvenient risks of healthcare efforts versus the benefits. Every part of the process is good to optimize. The use of small-diameter needles for the implantation of fiducial markers seems to reduce the rate of infections.
